# Efficient deformation algorithm for plasmid DNA simulations

**DOI:** 10.1186/1471-2105-15-301

**Published:** 2014-09-15

**Authors:** Adriano N Raposo, Abel JP Gomes

**Affiliations:** Instituto de Telecomunicações, Universidade da Beira Interior, Covilhã, Portugal, Av. Marquês Dávila e Bolama, 6200-001 Covilhã, Portugal

**Keywords:** Plasmid DNA, Simulation, Monte Carlo

## Abstract

**Background:**

Plasmid DNA molecules are closed circular molecules that are widely used in life sciences, particularly in gene therapy research. Monte Carlo methods have been used for several years to simulate the conformational behavior of DNA molecules. In each iteration these simulation methods randomly generate a new trial conformation, which is either accepted or rejected according to a criterion based on energy calculations and stochastic rules. These simulation trials are generated using a method based on crankshaft motion that, apart from some slight improvements, has remained the same for many years.

**Results:**

In this paper, we present a new algorithm for the deformation of plasmid DNA molecules for Monte Carlo simulations. The move underlying our algorithm preserves the size and connectivity of straight-line segments of the plasmid DNA skeleton. We also present the results of three experiments comparing our deformation move with the standard and biased crankshaft moves in terms of acceptance ratio of the trials, energy and temperature evolution, and average displacement of the molecule. Our algorithm can also be used as a generic geometric algorithm for the deformation of regular polygons or polylines that preserves the connections and lengths of their segments.

**Conclusion:**

Compared with both crankshaft moves, our move generates simulation trials with higher acceptance ratios and smoother deformations, making it suitable for real-time visualization of plasmid DNA coiling. For that purpose, we have adopted a DNA assembly algorithm that uses nucleotides as building blocks.

## Background

Plasmid DNA (pDNA) is a family of DNA molecules widely used in life sciences, more specifically in gene therapy research. These molecules are produced inside host cells in a supercoiled conformation (i.e., their natural conformation), which is the desired conformation for therapy purposes. However, such molecules can lose their original conformation in the production and purification processes, assuming more relaxed or even linear conformations, owing to thermodynamic changes (e.g., temperature changes). One of the main challenges for researchers is to find optimal thermodynamic conditions for plasmid DNA therapeutic application without losing its supercoiled conformation or, at least, minimizing the occurrence of relaxed or open DNA molecules.

For many years, computational methods based on laboratory experimental data have been proposed to model and simulate the dynamic behavior and conformational changes in pDNA molecules under certain conditions. The Monte Carlo (MC) method has generally been accepted as a reliable tool for simulation purposes, and is seen as the standard. This iterative method tries to minimize the elastic energy of the molecule in each iteration step of the simulation process, testing the probability of acceptance of each new trial. The goal is to make the molecule converge to an equilibrium state after performing as few iterations as possible, i.e., maximizing the acceptance ratio of the trials without compromising the effectiveness and reliability of the simulation.

To simplify the simulation process, each plasmid DNA molecule is reduced to a linear skeleton (i.e., polyline) with equal sized segments that represents the topological conformation of the molecule. Random deformations are then applied to this skeleton, generating new trial conformations, which are either accepted or rejected. Interestingly, the essence of the method used to randomly generate each new trial, referred to as the *standard* crankshaft move, has remained the same for many years, with its origins dating back to the early 1960s [1–3], more specifically in the context of lattice polymer chains. This move was later adapted for simulation of flexible molecules like DNA using MC methods.

However, the standard crankshaft move has a very low acceptance ratio of trials, i.e., many trials are rejected. Moreover, it can present very unnatural behavior as it features very sudden motions along large portions of the molecule. To enhance the efficiency of MC moves, biasing was found to be a solution [4, 5]. However, as Earl and Deem noted [6], biasing a deformation move implies that the probability of moving from one state to another is no longer symmetric; consequently, the acceptance rule used must be altered to maintain the detailed balance.

In this paper, we present a new unbiased move for plasmid DNA, whose skeleton is a closed polyline. This move not only preserves the size of each segment and its connectivity, but is also very effective in maximizing the acceptance ratio of the trials and stabilizing the molecule, thereby allowing steady, gradual temperature changes during the simulation. Our method also generates natural and realistic animations that can be used in real-time simulation and visualization.

## Related work

In this section, we briefly review the MC methods in computational biology and chemistry, as well as the generative methods for DNA conformations that form the core of these MC methods.

### Monte Carlo simulations

The MC simulation method is one of the most important methods used in DNA simulations. This method, which was originally presented by Metropolis et al. [7], generates DNA conformations combining energy calculations, random conformational changes, and statistics.

Frank-Kamenetskii et al. [8], Vologodskii et al. [9] and Lebret [10] were the first to use an MC method to present numerical results of the probability of the occurrence of knots on pDNA. Frank-Kamenetskii and Vologodskii also presented valuable information on DNA torsional rigidity [11]. A few years later, Vologodskii et al. used MC simulations to study the conformational and thermodynamic properties of DNA molecules with physiological levels of supercoiling [5]. Vologodskii also included a chapter on “Monte Carlo Simulation of DNA Topological Properties” in the book “Topology in Molecular Biology” [12], and with Rybenkov, they reviewed how conformational properties of DNA catenanes can be studied using MC simulations [13].

Gebe et al. [14] presented an MC algorithm to simulate supercoiling free energies in unknotted and trefoil knotted inextensible circular chains with finite twisting and bending rigidity, while Marko et al. [15] made use of MC simulations to study the relationship between the amount of twisting in DNA molecules and its supercoiling.

Kundu et al. used an MC algorithm to explain denaturation characteristics in a supercoiled plasmid and calculate the probability of denaturation for each base pair at different supercoiling degrees [16].

In their work on the relationship between knots and supercoiling, Cozzareli et al. used an MC simulation procedure to generate an equilibrium set of conformations [17].

MC simulations have also contributed to the understanding of the interplay between base-pair stacking interaction and permanent hydrogen-bond constraints in supercoiled DNA elasticity [18].

Based on the fact that atomic force microscopy has generated images of supercoiled DNA confined to a surface, which affects conformational properties such as twist and writhe, Fujimoto and Schurr modified an existing program, developed to perform MC simulations of supercoiled DNA in solution, flattening the DNA to simulate the effect of deposition on a surface [19]. Fujimoto and Schurr also presented a method to estimate torsional rigidities of weakly strained DNA [20].

Burnier et al. used MC calculations to identify a mechanism by which topoisomerases can keep the knotting level low [21].

More recently, Olson et al., in their paper “How stiff is DNA?”, used MC simulations to understand the behavior of a long, double-helical polymer in the tight confines of a cell and in the design of novel nanomaterials and molecular devices [22].

### Generative methods of DNA conformations

It has generally been accepted that supercoiled, i.e., the self twisting of the double stranded molecule over itself, is the desired conformation for pDNA molecules [23]. Thus, it is necessary to measure the supercoiling of a given molecule. One of the most important quantitative measures of closed circular DNA supercoiling is the linking number (*Lk*) of the two DNA strands, which is an integer corresponding to the number of double-helical turns of the molecule.

There are several methods for calculating *Lk*, with one of the most widely used involving the computation of two very important geometrical properties of closed circular DNA molecules: twist (*Tw*) and writhe (*Wr*). *Tw* features the coiling of the two DNA strands around the axis of the helix, while *Wr* is a measure of the coiling of the helix axis in space [24]. Thus, the main result is:


In our implementation, we used Klenin and Langowski’s computation method (2a) to calculate *Wr* [25].

Owing to the nature of three-dimensional closed polylines, knots can occur in some pDNA conformations. This is not a desirable feature, i.e., each closed circular DNA molecule must remain unknotted during the simulation process, keeping its original topology even if supercoiling occurs. Knot detection methods must be used during simulation to reject possible knotted conformations. We adopted Harris-Harvey’s knot detection algorithm, which uses the Alexander polynomial to detect the existence of knots [26]. This algorithm is based on the predicate that if two knots have different Alexander polynomials, then the knots are topologically distinct. Thus, because the Alexander polynomial of an unknotted closed circular DNA molecule is equal to one, all conformations for which this polynomial does not equal one must be rejected during the simulation.

**Figure 1:**
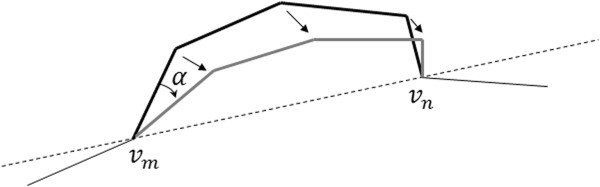
**Crankshaft motion.**

Each trial conformation must be generated in such a way that the size and connectivity of each segment of the DNA chain do not change. A major deformation method used to displace vertices of the DNA chain was introduced by Klenin et al. [4, 5]. This method, which is just a biased crankshaft move, starts by randomly choosing two vertices *v*_*m*_ and *v*_*n*_. Then, all the vertices (and consequently all connecting segments) are rotated a randomly selected angle *θ* around the axis defined by the line connecting *v*_*m*_ and *v*_*n*_, as shown in Figure 1. Furthermore, following Klenin et al. [4], the value of *θ* is uniformly distributed over a certain interval, and must be continuously adjusted during the simulation to guarantee that about half the steps are accepted.

**Figure 2:**
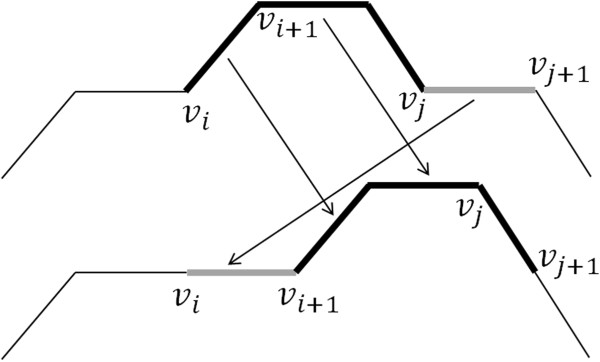
**Reptation motion.**

To increase the acceptance ratio of the simulation trials another type of motion has been proposed in the literature. This improvement performs a sub-chain translation, which is usually referred to as *reptation motion* [5], and is illustrated in Figure 2. First, two vertices *v*_*i*_ and *v*_*j*_ are randomly chosen. Then, the sub-chain between *v*_*i*_ and *v*_*j*_ is translated by one segment length along the chain contour. The segment that was immediately after *v*_*j*_ is also translated to fill the gap between *v*_*i*_ and *v*_*i*+1_. This motion suggests movement analogous to a snake slithering and, hence, the name reptation motion. Other types of motion can be adopted if the Metropolis microscopic reversibility requirement is satisfied, i.e., if the probability of each trial conformation is the same as that of the reverse movement [7].

Visualization of DNA conformational changes over time is also important as part of the entire simulation of DNA behavior. This is usually performed only when the entire simulation procedure ends and is typically done by assembling the DNA atoms along the DNA axis. Interestingly, a more efficient DNA assembly algorithm was presented to allow the visualization of all the steps of the simulation procedure in real-time [27]. In this method, each DNA nucleotide is represented by a three-dimensional building block, allowing the assembly of the entire molecule faster, but in a realistic way. In geometric terms, each of the four building blocks featuring DNA nucleotides is a Gaussian isosurface, which was previously generated by an algorithm that triangulates molecular surfaces [28].

## Methods

The deformation algorithm presented in this paper uses a linear skeleton (i.e., a polyline) with equal sized segments, henceforth called the DNA skeleton. Before introducing the core of the method itself, we explain how the DNA skeleton can be created for use by the deformation algorithm.

### Initial conformation of the DNA skeleton

The DNA skeleton can assume any closed unknotted conformation. The simplest of these conformations is a completely relaxed circular conformation. Besides, the length of each segment of the DNA skeleton corresponds approximately to 30 base pairs of the double helix [12].

That said, the first step of the algorithm is to determine the number of segments of the DNA skeleton ensuring around 30 base pairs per segment. Assuming that the DNA has a sequence of *n* base pairs, we want to find an integer *s* denoting the number of segments of the DNA skeleton. We define two integer parameters *min* and *max*, respectively, as the minimum and maximum numbers of base pairs that are admissible per segment, such that *min* < 30 <*max*. Then, for each integer value *i*,*min* ≤ *i* ≤ *max*, we calculate the corresponding *s*_*i*_=round(*n*/*i*). Finally, we adopt *s*=*s*_*i*_ as the number of segments of the DNA skeleton that minimizes |*n*-(*s*_*i*_. *i*)|.

Once we have the number of segments *s*, we just have to build a regular polygon with *s* sides inscribed in a circle. From the number of base pairs, we can infer the approximate perimeter of the circle, as well as the corresponding radius *R*, from which we obtain the first vertex of the skeleton at *p*_0_=(*R*,0,0). Then, we apply *s* successive rotations to *p*_0_ about the origin to obtain all the vertices of the DNA skeleton of the initial relaxed conformation; the rotation angle is given by *α*=2*π*/*s*. Note that, although the initial conformation is circular, the methods for DNA assembly and deformation apply to any initial conformation.

### Skeleton deformation algorithm

Assuming we have a three-dimensional closed polyline **P**_*k*_ representing the DNA skeleton, we need to deform this polyline to obtain a new polyline **P**_*k*+1_ with the same number of equal sized segments as **P**_*k*_, but without loss of its connectivity.

**Figure 3:**
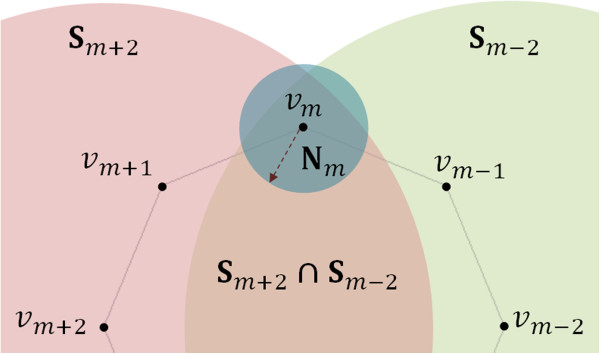
***Mobile vertex*** **v**_***m***_ **can be displaced randomly in the intersection of three spheres,** **N**_***m***_ **,** **S**_***m*-2**_, and **S**_***m*+2**_ **.**

Let *s* be the number of segments of **P**_*k*_ and {**v**_*i*_}, *i*=0,…,*s*-1, its vertices. We choose a random vertex **v**_*m*_, 0≤*m*≤*s*-1 as our *mobile vertex*, i.e., the vertex with the most motion freedom in the current trial conformation. Any movement of the *mobile vertex***v**_*m*_ implies movement of its closest neighbors **v**_*m*-1_ and **v**_*m*+1_, called *semi-mobile vertices* (Figure 3). The remaining neighbors **v**_*m*-2_ and **v**_*m*+2_ are *fixed vertices* because they do not move in the deformation. Thus, in each deformation step, only three vertices will be displaced: **v**_*m*_, **v**_*m*-1_ and **v**_*m*+1_.

However, **v**_*m*_ cannot be freely displaced (Figure 3). In the first instance, **v**_*m*_ moves within the sphere **N**_*m*_ centered at **v**_*m*_ with radius *r*=2 *Δ*, where *Δ*=3.3Å is the distance between two consecutive base pairs. More specifically, the new position of **v**_*m*_ is found randomly within the region resulting from the intersection of the three spheres, **N**_*m*_, **S**_*m*-2_ and **S**_*m*+2_. The latter two spheres with radius 2*l* are centered on the fixed vertices **v**_*m*-2_ and **v**_*m*+2_, respectively, where *l* is the length of each segment of the DNA skeleton. Note that the optimal value 2 *Δ* for *r* was found experimentally and based on the rate of successful trial conformations in the first attempt, about 30 percent, though this rate remained high for a value of *r* up to 3 *Δ*. The small radius *r* of sphere **N**_*m*_ ensures that the transition from **P**_*k*_ to **P**_*k*+1_ occurs without noticeable jumps.

As noted above, the new position of **v**_*m*_ was found randomly within the region **N**_*m*_∩**S**_*m*-2_∩**S**_*m*+2_ (Figure 3), but no explanation of this random procedure was given. In fact, to calculate the new position of **v**_*m*_, we first convert its Cartesian coordinates (*x*,*y*,*z*) to spherical coordinates (*d*,*θ*,*ϕ*) relative to **v**_*m*-2_, where *d* is the distance between **v**_*m*-2_ and **v**_*m*_. Next, we randomly generate a new position for **v**_*m*_ as (*d*+*Δd*,*θ*+*Δθ*,*ϕ*+*Δϕ*), where *Δd*∈[-*r*,*r*] and *Δθ*,*Δϕ*∈[-*π*,*π*]. It is clear that the displacement of the flanking vertices **v**_*m*-1_ and **v**_*m*+1_ depends on the previous movement of **v**_*m*_. Here we focus on the computation of the new position of **v**_*m*-1_ since the new position of **v**_*m*+1_ can be calculated similarly.

**Figure 4:**
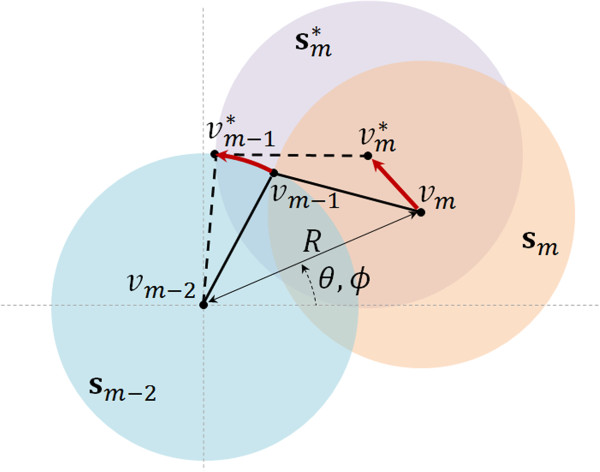
**Displacement of vertices v**_***m***_ **and** **v**_***m*-1**_ **.**

**Figure 5:**
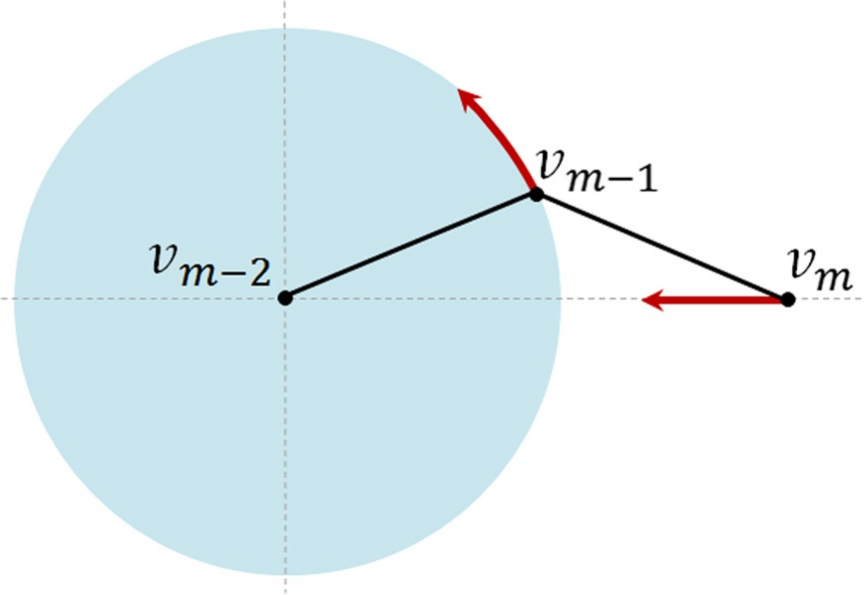
**Translational piston move of vertex v**_***m***_ **translates into a rotational move of v**_***m*-1**_ **.**

For this purpose, we also convert the Cartesian coordinates of **v**_*m*-1_ to spherical coordinates (*l*,*α*,*β*) relative to **v**_*m*-2_, where *l* is the radius of the three spheres **s**_*m*_, **s**_*m*-1_, and **s**_*m*-2_ centered on **v**_*m*_, **v**_*m*-1_, and **v**_*m*-2_, respectively. Moving **v**_*m*-1_ to a new position must be done in such a way that its distance *l* to **v**_*m*-2_ and **v**_*m*_ remains unchanged. In other words, the new **v**_*m*-1_ must lie on the circumference resulting from the intersection of the two surfaces bounding **s**_*m*_ and **s**_*m*-2_ (Figure 4). If *Δd*=0, the new position of **v**_*m*-1_ relative to **v**_*m*-2_ is given by (*l*,*α*+*Δθ*,*β*+*Δϕ*); otherwise, the new location of **v**_*m*-1_ is (*l*,*α*+*Δθ*+*Δψ*,*β*+*Δϕ*), where *Δψ* is the angle of the angular motion of **v**_*m*-1_ on **s**_*m*-2_ resulting from the translational displacement *Δd* of **v**_*m*_ along the line defined by **v**_*m*_ and **v**_*m*-2_ (Figure 5). We compute *Δψ* by rearranging the equation that describes the reciprocal motion of the piston with respect to the crank angle as follows (cf. [29], p.44):
1

Note that applying the translational displacement *Δd* to **v**_*m*_ before the rotational motions *Δθ* and *Δϕ* means that *Δθ*=0 in Eq. (1); otherwise, *Δθ*≠0. In summary, moving **v**_*m*_ implies a translational and two rotational motions relative to **v**_*m*-2_ expressed in spherical coordinates. This causes **v**_*m*-1_ to rotate accordingly on the sphere centered at **v**_*m*-2_, with part of this rotational motion determined by the translational displacement *Δd* of **v**_*m*_.

These types of moves (i.e., translation and rotation) satisfy the principle of microscopic reversibility [30], although this is not critical for our purposes because the simulation procedure is only used to locate energy minima. As noted by Mauri in [31], for a conservative *n*-body system, as in the case of a DNA molecule, microreversibility stems from the invariance of the equations of motion with respect to time reversal, i.e., every microscopic motion reversing all particle velocities also results in a solution. This leads to the so-called principle of detailed balance [32], which states that under stationary conditions (i.e., all probability distributions are invariant under time translation) each possible transition from one conformation to another balances itself with the reversed transition in time. In other words, the probability of obtaining trial conformation **P**_*k*+1_ if the current conformation is **P**_*k*_ must be equal to the probability of obtaining trial conformation **P**_*k*_ if the current conformation is **P**_*k*+1_ [5].

With this in mind, and having calculated the constrained position of **v**_*m*-1_ as a consequence of the move of **v**_*m*_, we need to determine its new position after rotating it randomly about the axis defined by **v**_*m*-2_ and **v**_*m*_. It is clear that the old and new locations of **v**_*m*-1_ lie on the circumference resulting from the intersection of spheres **s**_*m*-2_ and **s**_*m*_. Likewise, we find the new position of **v**_*m*+1_ after rotating it randomly about the axis defined by **v**_*m*+2_ and **v**_*m*_. Interestingly, these two rotations can be seen as two particular crankshaft rotations.

Finally, it is worth noting that the deformation algorithm described above can also be used in other biochemical systems such as internal coordinate models of cyclic peptides, as well as in some mechanical problems related to articulated arms and chain moves. In fact, this algorithm can be used to randomly deform any regular polygon (or polyline with equal sized segments) in two or three dimensions with guaranteed preservation of connectivity and the length of segments.

### DNA assembly algorithm

**Figure 6:**
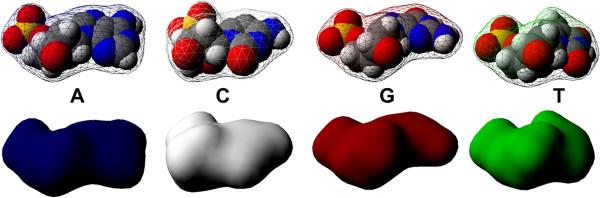
**DNA building blocks: (A) adenine, (C) cytosine, (G) guanine, and (T) thymine.**

For a realistic visualization of closed circular DNA simulations in real-time, we combine the new deformation algorithm described above with the DNA assembly algorithm introduced by Raposo and Gomes [27]. This DNA assembly algorithm uses four three-dimensional building blocks representing DNA nucleotides (Figure 6), namely, adenine (A), cytosine (C), thymine (T), and guanine (G). Each building block is a pre-triangulated isosurface generated by a triangulation algorithm for molecules [28]. The assembly procedure for nucleotides can be thought of as the operation of wrapping helicoidal DNA backbones around cylinders along the DNA skeleton. The algorithm iterates over *N* base pairs of the DNA, assembling a single base pair *b*_*i*_*B*_*i*_ per iteration. Each iteration comprises four distinct stages:

- *Generation of geometric instances for nucleotidesb*_*i*_*andB*_*i*_*.* Considering that there are only four possible base pairs, given a nucleobase *n*_*i*_ of a DNA strand, two geometric instances of nucleotides must be generated, the first for the building block *b*_*i*_ and the second for the corresponding building block *B*_*i*_.
- *Positioning of the base pairb*_*i*_*B*_*i*_*on planez*=0. Base pair *b*_*i*_*B*_*i*_ is positioned as if it was the first base-pair of the DNA molecule, that is, it is placed perpendicular to the segment that starts at the origin in such a way that its center coincides with the origin.
- *Alignment of base pairb*_*i*_*B*_*i*_*with the plane perpendicular to segment i.* Note that this alignment involves a rotation about the origin of the coordinate system.
- *Translation of base pairb*_*i*_*B*_*i*_*to the plane perpendicular to segment i.* Finally, because all geometric transformations (i.e., translations and rotations) are performed around the origin, building blocks *b*_*i*_ and *B*_*i*_ must be displaced to their correct positions relative to the midpoint of the corresponding segment *i* of the DNA axis.

**Figure 7:**
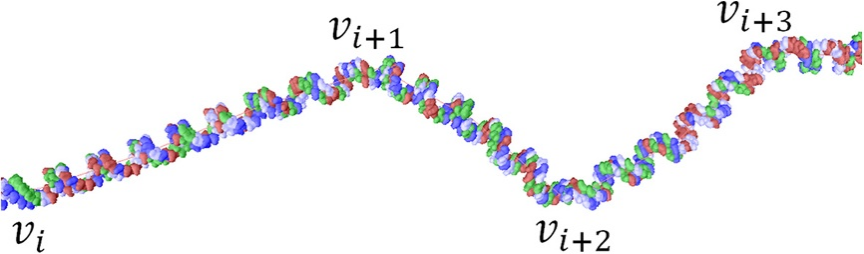
**Detail of DNA segments assembled around a random piece of the skeleton.**

It is important to note that this DNA assembly algorithm does not take into account the sharp kinks that may occur at the junctions of the conformation segments, as shown in Figure 7. Nevertheless, a possible solution to this problem is the smoothing procedure proposed by Kummerle and Pomplun [33]. For detailed information about the DNA assembly algorithm, the reader is referred to [27].

### Monte Carlo simulation

MC simulations are iterative methods based on the concept of *elastic energy* of closed circular DNA and on stochastic parameters and calculations aimed at converging to the energetic and thermodynamic equilibrium of the molecule. The main principle is to perform random DNA deformations and check whether the resulting new conformations should be accepted or rejected according to energy changes and acceptance probability. More specifically, a random deformation of the DNA is accepted if it reduces the *elastic energy* of the molecule or has some probability of occurring. In the experiments and results presented in this paper, we used the same MC simulation method and parameters as those used in [33], where *elastic energyE* is calculated as
2

Here *E*_*b*_ is the *bending energy* given by
3

where *k*_*B*_ is the Boltzmann constant, *T* is the temperature, *α*=2.403 is the bending constant, and *Θ*_*i*_ is the angular displacement between the directions of segments *i* and *i*+1. *Torsional energyE*_*t*_ is given by
4

where *C* denotes a constant parameter known as the torsional rigidity, *L* is the total length of the chain, and *Wr* is the writhe of the skeleton. The linking number difference *ΔLk* in (4) is the difference between the linking number *Lk* of the DNA molecule and that, *Lk*_0_, of its relaxed DNA conformation
5

where -0.07≤*σ*≤-0.05 is the superhelix density parameter [34].

For calculating writhe *Wr*, we used the method proposed in [25], more specifically, method 2b [10]. This method is based on the principle that writhe can be calculated as the difference between linking number *Lk* and twist *Tw*:
6

This method for computing *Wr* uses an auxiliary chain close enough to the DNA skeleton, and with as many segments *s*_*i*_ as the DNA skeleton. Then, considering that *r*_*i*_ is the initial point of segment *s*_*i*_, we can find the directional writhe as follows:
7

where
8

For the computation of (8), we must check whether segments *s*_*i*_ and *s*_*j*_ cross, i.e., whether their projections *π*(*s*_*i*_) and, *π*(*s*_*j*_) onto plane *z*=0 intersect. We used LaMothe’s algorithm to check whether the projections of these segments intersect [35].

In turn, and following Klenin and Langowski [25], *twist* is given as
9

where **p**_*i*_ denotes the vector, normal to both *s*_*i*-1_ and *s*_*i*_, (**p**_*i*_)_*z*_ denotes the *z*-th component of vector **p**_*i*_ vector, and
10

where **u** is the unit vector in the *z* axis direction.

Then, using the results of (7) and (9), we get the final *writhe* number:
11

Once we know how to perform the necessary energy calculations, we can apply the MC method to each iteration of the simulation to obtain a new DNA conformation from a random deformation of the DNA skeleton. Then, we calculate the energy *E*_*i*+1_ of the new candidate conformation, and compare it with the energy, *E*_*i*_, of the previous conformation. The new conformation *i*+1 is accepted if *E*_*i*+1_<*E*_*i*_ or
12

where *T*_*M*_ is the temperature of the experiment and *ρ* is a random value between 0 and 1 [33].

### Knots detection

It is important to note that knots can occur when random deformations are applied to DNA conformations. Because this is not desirable, i.e., DNA supercoiling must occur without generating knots, we must check for the existence of knots and reject the deformation if we find one or more knots. To optimize the performance of the method, this checking procedure is done before the MC acceptance test, avoiding unnecessary energy calculations.

For knot detection we used the method of Harris and Harvey [26]. In this method, based on the principle that two knots are topologically distinct if they have distinct Alexander polynomials, the DNA skeleton is converted to a knot data structure, and its Alexander polynomial is computed and compared with the Alexander polynomial of the circle, which is a trivial knot. If these two polynomials are different, the DNA skeleton contains at least one non-trivial knot.

## Results

To evaluate the effectiveness and performance of our deformation method when applied in MC simulations, we performed a set of experiments comparing our method with two types of DNA chain moves, namely, the *standard* crankshaft move and the *biased* crankshaft move.

The standard crankshaft move is a randomly chosen move. In fact, the ends, *v*_*m*_ and *v*_*n*_, of each sub-chain are randomly chosen, as is the case with the rotating angle *θ* of the sub-chain around the line that passes through its ends (cf. Figure 1). That is, the standard crankshaft move does not adjust the size of the sub-chain nor the rotation angle in any way. On the other hand, as in the deformation method introduced by Klenin et al. [4, 5], the biased crankshaft move used here adjusts only the rotation angle. Recall that this type of biased moves is a way of enhancing the efficiency of MC moves, because it allows us to choose moves with a higher acceptance ratio [6].

Through these experiments we aim to demonstrate that our method generates a smoother and more controlled deformation, which leads to more consistent and even faster convergence to molecular energy equilibrium.

### Experimental setup

Three experiments (A, B, and C) were performed to compare the proposed method with two classic methods, namely, the standard crankshaft move and biased crankshaft move.

We used a setup based on Kummerle and Pomplun’s work [33] for the pUC19 plasmid DNA molecule. All the three experiments were performed using the same closed circular DNA sequence with 2686 base pairs (pUC19 [36]) and exactly the same conditions and MC simulation parameters for the three Monte Carlo moves under comparison, that is: *k*_*B*_=1.38^-23^; *α*=2.403; *C*=3×10^-^28; and *σ*=-0.04. However, we performed experiment A at a constant temperature of 293 K, while experiments B and C were performed progressively reducing the temperature from 350 K to 10 K.

Finally, it should be noted that the MC simulations were performed on an 64-bit Windows 7 laptop computer with an Intel i5 2.40 GHz CPU, 4 GB RAM and an Nvidia Geforce GT 520 MX 1 GB graphics card.

### Experiment A: pUC19 with constant temperature

**Figure 8:**
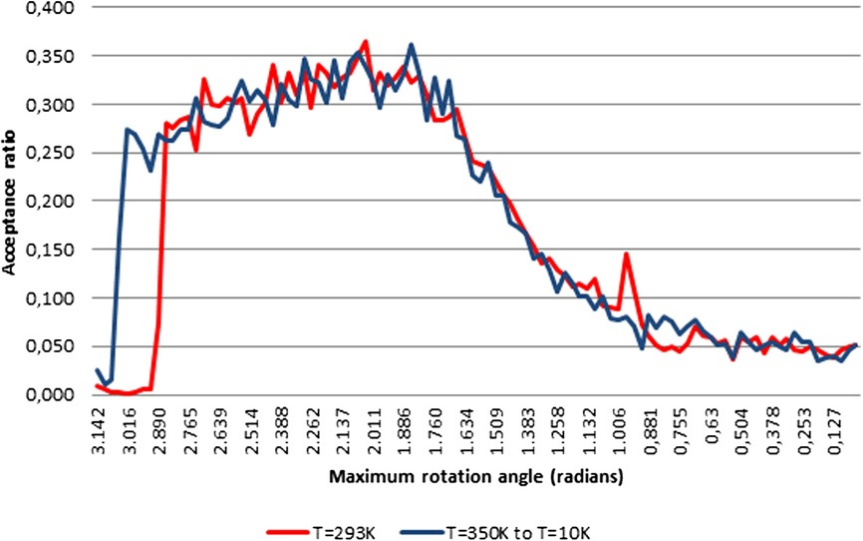
**Optimization of initial angle range for 100,000 steps.**

**Figure 9:**
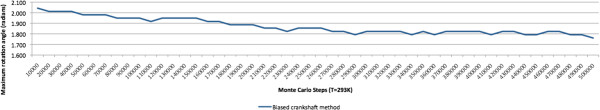
**Experiment A: Crankshaft rotation angle for 500,000 steps at a constant temperature of 293 K.**

In experiment A, we performed an MC simulation with 500,000 steps using the pUC19 closed circular DNA sequence at a constant temperature of 293 K as in [33]. This experiment was replicated using: (a) the standard crankshaft move, (b) the biased crankshaft move as described in [4, 5], and (c) the proposed method. In the particular case of the biased crankshaft move, after an exhaustive optimization procedure with more than 100,000 steps, we came to the conclusion that the crankshaft rotation angle should initially be in the range [-2.043,2.043] (radians), as shown in Figure 8. Furthermore, as expected, this angle decreases over time Figure 9.

**Figure 10:**
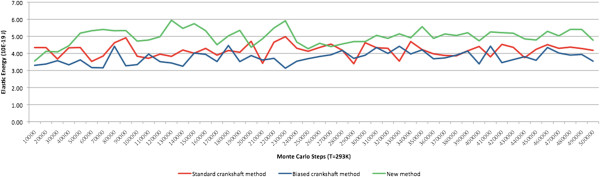
**Experiment A: Elastic energy for 500,000 steps at a constant temperature of 293 K.**

We used two measures to compare the efficiency of the three methods: (1) *elastic energy equilibrium* and (2) *acceptance ratio* of trials. The graphs of *elastic energy* for the three methods are shown in Figure 10, where we can see that the average elastic energy for each method remains approximately the same over time. Nevertheless, on average, the elastic energy of the proposed method is slightly higher than that of the standard crankshaft method, which in turn is higher than the energy associated with the biased crankshaft method.

**Figure 11:**
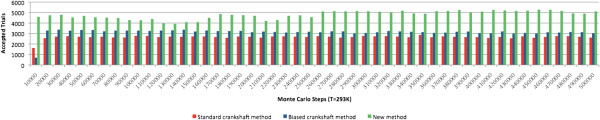
**Experiment A: Acceptance trials for slices of 10,000 steps from a total of 500,000 steps at a constant temperature of 293 K.**

On the other hand, the *acceptance ratio* of trials was evaluated for each slice of 10,000 steps from a total of 500,000 steps (see Figure 11), according to the acceptance condition (12). The acceptance ratio was steadily higher for the proposed method, always remaining above 4,000 (and even reaching 5,000) accepted steps for each slice of 10,000 steps, i.e., an average acceptance ratio around 45%, and achieving higher ratios around 50% in the second half of the experiment. On the contrary, the acceptance ratio for the standard crankshaft move was always under 30%, and even lower in the first 10,000 steps of the experiment. With respect to the biased crankshaft move, the average acceptance ratio was slightly above 30%, but far below the results obtained using the proposed method. This indicates that our new method generates trials with much higher probabilities of being accepted under the MC simulation conditions at a constant temperature, i.e., it minimizes the number of trial rejections, and avoids useless computations.

During experiment A, we also noted that the crankshaft moves generated a few hundred conformations that were rejected owing to the existence of knots. On the contrary, our new method did not produce any knots at any time during the 500,000 simulation steps because the deformation is done smoothly and without conformational jumps. This concurs with the fact that, despite DNA molecules in living cells being long and compactly coiled, they rarely get knotted [21], which suggests that supercoiling inhibits DNA knotting.

### Experiment B: pUC19 with variable temperature

**Figure 12:**
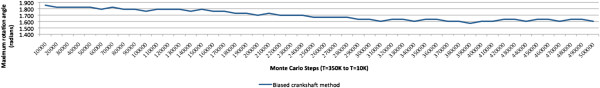
**Experiment B: Crankshaft rotation angle for 500,000 steps with temperature varying between 350 K and 10 K.**

**Figure 13:**
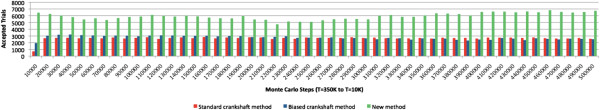
**Experiment B : Acceptance trials for slices of 10,000 steps from a total of 500,000 steps with temperature varying between 350 K and 10 K.**

Experiment B also involved 500,000 MC steps. This experiment was also replicated for each of three methods analyzed in this paper. In the particular case of the biased crankshaft move, once again, after an exhaustive optimization procedure with over 100,000 steps, we concluded that the initial crankshaft rotation angle should be in the range [ -1.854,1.854] (radians) because, in experiment A, the temperature is not constant. Adjustments to this rotation angle range during the experiment are shown in Figure 12.More specifically, the temperature decreases with energy, i.e., if the average elastic energy of a 1,000-step interval is higher than that of the previous slice of 1,000 steps multiplied by a 0.9 factor, the temperature is also multiplied by a 0.9 factor. In fact, the temperature decreased progressively from 350 K to 10 K. As expected, the closer the method converges to the energy equilibrium, the greater is the decrease in temperature. As in experiment A, the acceptance ratio of the proposed deformation method was always higher than those of the classic deformation methods (see Figure 13).

**Figure 14:**
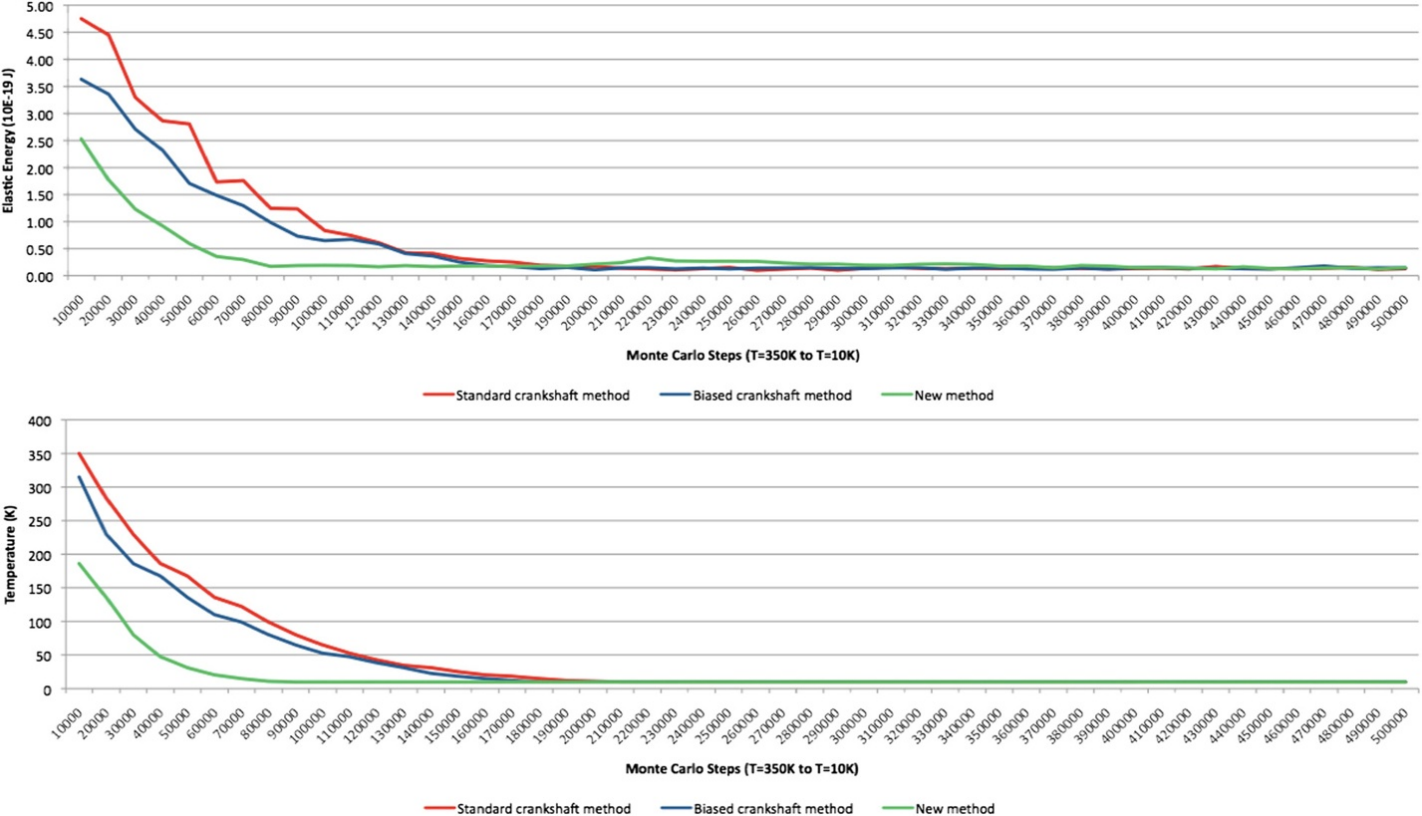
**Experiment B: (top) elastic energy during a 500,000-step experiment with temperature varying between 350 K and 10 K; (bottom) temperature decaying during a 500,000-step experiment.**

**Figure 15:**
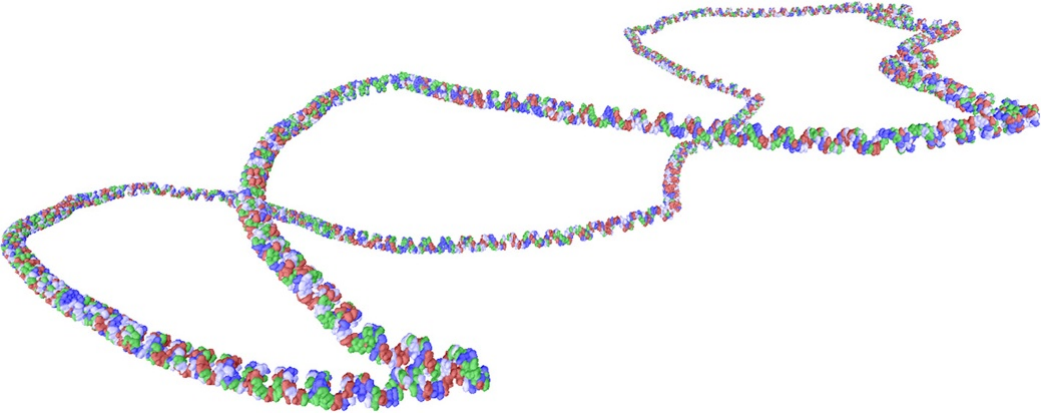
**Experiment B: pUC19 after 350,000 MC steps with temperature varying between 350 K and 10 K.**

It was a somewhat surprising to observe how the energy decreased during the simulation. As shown in Figure 14 (top), when using the proposed method, the elastic energy of the molecule converged sooner and more consistently to equilibrium. As shown, we achieved energy equilibrium after approximately 80,000 MC steps, while the crankshaft moves only stabilized after 160,000 steps. Besides, the standard crankshaft move generated a number of very slight energy jumps, i.e., the energy did not decay as consistently as in the proposed method. However, the energy level at equilibrium was the same for all three methods, approximately 0.14×10^-19^.No less meaningful was the temperature decay during this experiment. As presented in Figure 14 (bottom), when using the proposed method, the MC temperature decreased more rapidly and in a more consistent way, i.e., the graph for the new method is much smoother with the advantage of reaching the equilibrium temperature sooner. Figure 15 shows the final result of experiment B.

In summary, we can say that the proposed deformation method requires fewer simulation steps to achieve energy equilibrium, largely owing to its high acceptance ratio.

### Experiment C: average displacement

**Figure 16:**
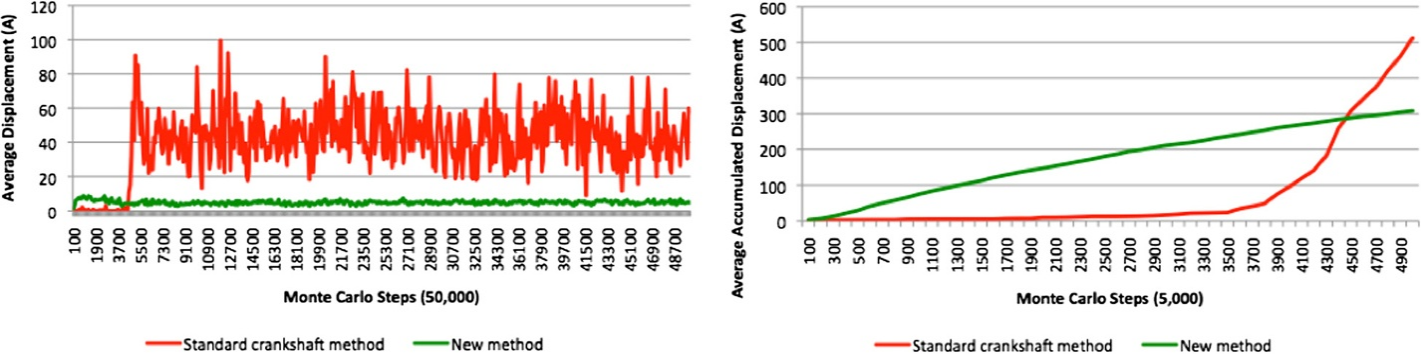
**Experiment C: (left) average displacement per 100 steps for pUC19 50,000 steps; (right) average accumulated displacement for pUC19 5,000 steps.**

In experiment C, we set out to measure the amount of deformation of plasmid DNA caused by each type of MC move. This was accomplished by computing the average displacement of the DNA skeleton vertices for each accepted trial. In this experiment, we only considered the standard crankshaft move and the move proposed in this paper. Taking into account vertices *v*_*i*_,*v*_*i*+1_,…,*v*_*i*+*n*_ that are displaced during a simulation trial, we determined the distances *d*_*i*_,…,*d*_*i*+*n*_ between the new positions and the previous positions of these vertices, and straightforwardly computed the average displacement given by (*d*_*i*_+…+*d*_*i*+*n*_)/(*n*+1). Finally, we considered the accumulated displacement for a slice of steps as the sum of the average displacements of the accepted trials of that slice.More specifically, we performed a 5,000-step simulation for each of the methods, namely, the standard crankshaft move and the proposed move. As shown in Figure 16 (left), the new move generates much smaller average displacements than the standard crankshaft move. Besides, from Figure 16 (right) we can see that the new move generates displacements right from the start of the simulation, whereas the standard crankshaft move starts to produce displacements later. This can be explained by the high acceptance ratio of the new method, as well as its more steady deformations.Moreover, from Figure 16, we conclude that smaller displacements in each trial do not mean there will be smaller accumulated displacements. The accumulated displacements of the new method form a logarithmic curve, while the curve of the standard crankshaft move is clearly exponential (cf. Figure 16 (right)). This means that in the new method, the closer we get to the point of energy equilibrium, the shorter is the displacement toward a stable conformation, i.e., the average displacement for each accepted trial converges to zero as the conformation converges to the equilibrium. This is not the case in the standard crankshaft move, as illustrated by the accentuated variations in average displacement of the trials in Figure 16 (left).

### Discussion

As mentioned above, we used the same molecule (i.e., pUC19), the same conditions/parameters, and the same MC simulation method in experiments A, B, and C. For comparison purposes, each experiment was performed using three different deformation methods: (a) the *standard* crankshaft move, (b) the *biased* crankshaft move (i.e., with rotation angle optimization and adjustment), and (c) the proposed method.

As expected, the acceptance ratio of trials for the proposed method is higher than that for either of the crankshaft moves. The acceptance ratio of the new method is almost always greater than 40%, and even reaches more than 60% at certain times. Moreover, unlike the crankshaft moves, the acceptance ratio for the proposed method is very high from the very first steps of the simulation. More specifically, in a scenario with decreasing temperature, we obtained an acceptance ratio of more than 60% for the proposed method compared with 5% for the standard crankshaft move and 20% for the biased crankshaft move in the first 10,000 MC steps.

With respect to elastic energy, the experiments also show that as the temperature decreases the new move achieves better performance than either of the crankshaft moves. In fact, we noted that elastic energy tends to its equilibrium point not only in a smoother and more natural way, but also more quickly with fewer MC steps.On the other hand, with regard to the average displacement of vertices in each trial, which provides the deformation measure of each tentative conformation, we noted that, as expected, the proposed move produces smaller deformations than either of the crankshaft moves. However, the accumulated displacement of the proposed move is actually greater than those of both crankshaft moves in the first 4,000 or so simulation steps (cf. Figure 16 (right)). This high acceptance ratio in the initial simulation steps means that the proposed move generates a much more consistent deformation, the behavior of which obeys a logarithmic curve instead of the exponential curve that describes the accumulated deformation of each of the two crankshaft moves considered in this paper.

## Conclusion

The crankshaft rotation method is the most common move found in the plasmid DNA simulation methods for generating new DNA conformations. Recall that this classic method first selects two random vertices of the DNA skeleton, and then all the segments between these two vertices are rotated around the axis defined by them. This move is not very effective because many trials are rejected by the MC method. In addition to its low acceptance ratio, this method can generate unnatural movements with large portions of the DNA molecule displaced at once, unless the relevant parameters are appropriately adjusted.

In this paper, we introduced a new move for plasmid DNA through MC simulations. In each iteration of the simulation, only one vertex and its two closest flanking vertices are subjected to the motion procedure. Thus, for each new trial, a single vertex is randomly chosen and then randomly displaced to a point within a small neighborhood. To maintain connectivity of the DNA chain, as well as the size of its segments, the two flanking vertices are also displaced but in a less free way. Thus, only three vertices are displaced in each new trial.

Interestingly, considering that our algorithm generates small deformations in the transition from one DNA conformation to another, we can conclude that it can be applied not only in the simulation of DNA coiling, but also in real-time visualization. We have already done this by employing the DNA assembly algorithm that uses Gaussian surfaces as geometric representations of nucleotides, as mentioned at the end of Section ‘Related work’.

In the future, we intend to incorporate a smoothing mechanism into our DNA algorithm like, for example, that presented in [33]. This will enable our algorithm to produce even more realistic simulations, eliminating the occurrence of slightly sharp corners like those shown in Figure 15. We also intend generating deformations that depend on the DNA’s stiffness, which varies according to the sequence of nucleotides. This will mean greater deformations on more flexible segments and smaller deformations on less flexible segments of DNA.

Finally, our ultimate goal is to be able to replicate *in silico*, and visualize what happens to plasmid DNA during the production and purification processes in laboratory experiments.
